# Acute Limb Ischemia as a Concomitant Manifestation of COVID-19

**DOI:** 10.7759/cureus.21032

**Published:** 2022-01-08

**Authors:** Abdulaziz M Eshaq, Abdulsalam A Almofadhli, Noor K Aljarba, Mohammed A Karish

**Affiliations:** 1 Medicine, Alfaisal University, Riyadh, SAU; 2 Internal Medicine, King Salman Specialist Hospital, Hail, SAU

**Keywords:** covid-19, acute limb ischemia, coagulopathy, arterial thrombosis, sars-cov-2

## Abstract

Severe acute respiratory syndrome coronavirus 2 (SARS-CoV-2) is a novel virus that causes multi-systemic manifestations identified as coronavirus disease 2019 (COVID-19). Respiratory tract symptoms are the most commonly seen in infected patients with COVID-19. Hypercoagulability state is the most common coagulopathy disorder associated with critically ill COVID-19 patients. Several inflammatory and coagulation factors such as D-dimers and fibrinogen correlate with the degree of pro-thrombotic state and burden of the disease. We describe a case of a 51-year-old man who presented with respiratory pneumonia and concomitant severe bilateral arterial thrombosis followed by right above knee amputation.

## Introduction

Coronavirus disease 2019 (COVID-19) is a novel pandemic caused by severe acute respiratory syndrome coronavirus 2 (SARS-CoV-2) [1]. It has been first described as a cluster of cases in Wuhan, China lately in December 2019 [2]. Since its emergence, the number of cases has dramatically increased worldwide, over 278 million cases with a high case fatality of 4.5 million deaths have been reported as of December 26, 2021. In Saudi Arabia, as of the same date, the number of cases has reached 552,795 cases with 8,871 deaths [3]. The virus predominantly affects the respiratory system; however, it evolved to affect multi-organs and causes systemic and coagulation dysfunctions mainly hypercoagulability [4]. COVID-19 associated arterial thromboembolism is less evident; however, it has a poor prognosis [5]. Herein, we report an unusual case of bilateral lower limb severe ischemia due to acute arterial thrombosis in a 51-year-old man confirmed COVID-19 case, which resulted in right above knee amputation. To the best of our knowledge, it is the first case to be described in Saudi Arabia.

## Case presentation

This is a case of a 51-year-old male from Bangladesh who presented to emergency department complaining of fever, cough and shortness of breath since four days. There was no history of chest pain, palpitation, headache, abdominal pain, diarrhea or vomiting. The patient denied any contact with infected or suspected COVID-19 patient nor a history of recent travel. Past medical history was significant for diabetes and hypertension. His health conditions were well-controlled on metformin and amlodipine medications, and he was a non-smoker.

On examination, the patient’s vitals showed a temperature of 37.1 °C, blood pressure of 196/93 mmHg, heart rate of 102 beats per minute, and oxygen saturation of 73% on room air and 93% on 5 L. He was conscious, alert and oriented, However, he looks ill and distressed. Chest exam revealed bilateral crackles. The abdomen was soft, lax and non-tender.

**Figure 1:**
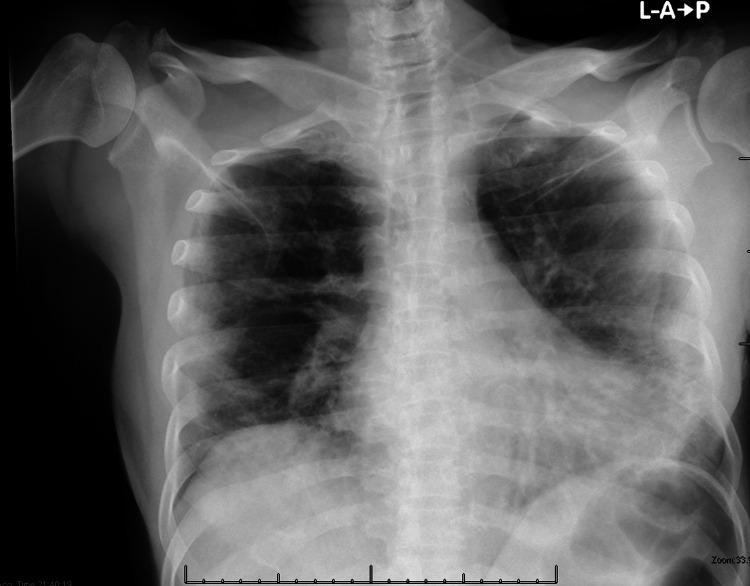
Chest X-ray Bilateral peripheral Infiltrate with predominantly basal scattered patchy opacities and consolidation seen more at the left lung.

The patient was admitted to the suspected COVID-19 isolation ward, and started on ceftriaxone, azithromycin and prophylactic enoxaparin. A nasopharyngeal swab was sent for a reverse transcriptase-polymerase chain reaction assay to assess for SARS-CoV-2 infection. Also, Chest X-ray was obtained and revealed bilateral lower lobe infiltrates consistent with respiratory pneumonia (Figure 1).

**Figure 2:**
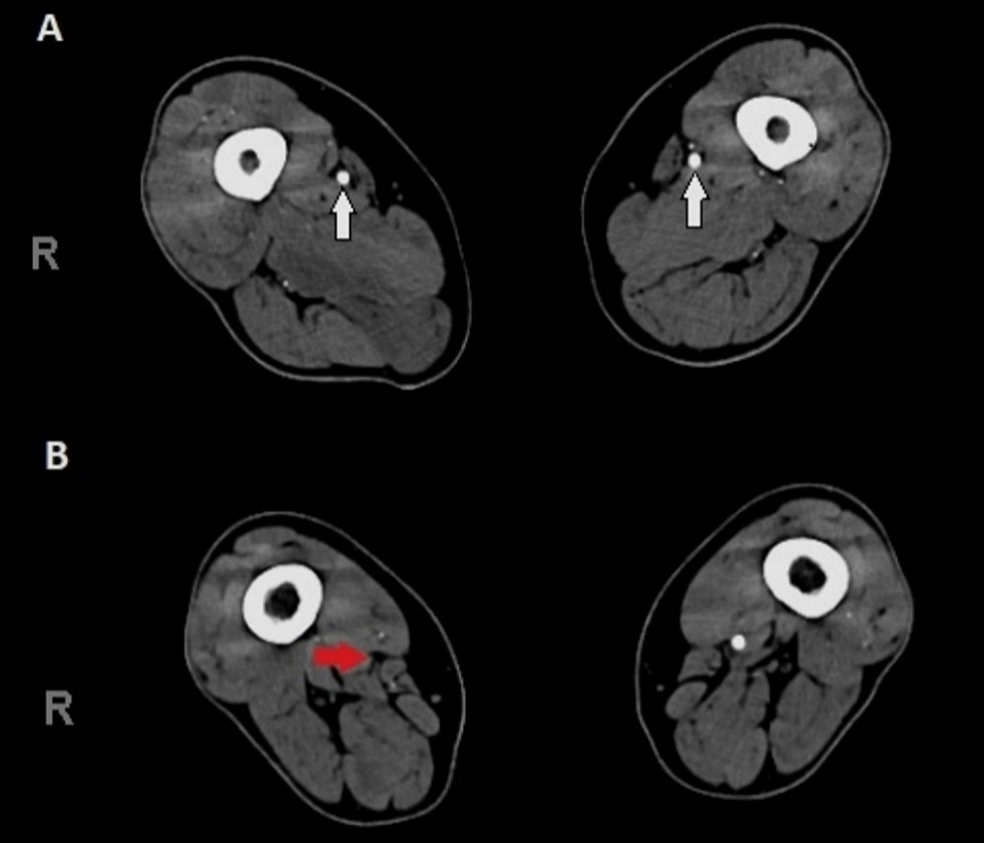
CT Angiography of lower limbs 2A, CT section showing normal contrast refilling at the level of the superficial femoral artery of both lower limbs (white arrows) 2B, Complete occlusion of the right lower third of superficial femoral artery and down arteries (red arrow)

Three hours post-admission, the patient experienced severe pain in the right lower limb. The patient denied any similar attacks of limb pain recently. On physical exam, the right leg was markedly pale, cold and weak with loss of sensation. The dorsalis pedis and popliteal pulses on the right leg were not palpable. Conversely, the examination of the left leg revealed some coldness, however, the sensation and movement were intact and skin changes were not present. Urgent vascular surgery consultation was made and ultrasound doppler was performed, which depicted extreme bilateral lower limb arterial insufficiency. Therefore, computed tomography (CT) angiography for both lower limbs was obtained which demonstrated atherosclerotic changes of the lower aorta and both lower limb arterial systems. Also, the lower abdominal aorta, left common iliac, and to a lesser extent, right common iliac had nearly total thrombotic occlusion. Also, there was multilevel arterial occlusion of both lower limbs affecting mainly infra-geniculate arteries. Most importantly, there was complete occlusion of the right lower third of superficial femoral artery (Figures 2A, 2B).

The patient has been reviewed by the vascular surgery team and according to their management plan, he is for conservative treatment. On the same day of admission, the patient’s oxygen requirement was increased as his oxygen saturation was 92% on 11 L. Therefore, according to the local COVID-19 treatment protocol, the patient was started on dexamethasone 6 mg intravenous once daily, aspirin 81 mg once daily, therapeutic dose of enoxaparin 5000 units every 12 hours.

Initial laboratory investigations demonstrated the following values (normal ranges are given in parentheses): hemoglobin 16 g/dL (males: 14-17 g/dL), white blood cells 15.4 K/mm^3^ (4500-11,000/mm^3^), platelets 380 K/mm^3^ (50-350 x 10^9^/L), neutrophils 89% (40% to 60%), lymphocytes 4.5% (20% to 40%), lactate dehydrogenase 1314 U/L (60-100 U/L), C-reactive protein 12.6 mg/L (0.0-8.0 mg/L) and erythrocyte sedimentation rate (ESR) was 70 mm/hr (0- 20 mm/hr) . Most importantly, D-dimers were extremely high at 61.6 µg/mL (<0.5 µg/mL). Also, potassium level was normal on admission 4.7 meq/L (3.5-5.0 meq/L). After five days the patient had a picture of rhabdomyolysis as a result of ischemia, the potassium level increased to 5.6 meq/L (3.5-5.0 meq/L) and creatine kinase (CK) was 3240 U/L (30-170 units/L). Coagulation profile were within normal values. Other labs such as blood urea nitrogen, creatinine were within normal limits.

On the second day of admission, the nasopharyngeal swab came positive for COVID-19, then he was shifted to COVID-19 confirmed cases ward. On hospital day three, his oxygen requirement was adjusted to 12 L and his labs showed high liver enzymes; high CK 36550 U/L, however, normal uric acid, phosphate, and calcium. Aggressive intravenous (IV) fluid of normal saline was given and the antibiotic was escalated to meropenem.

**Figure 3:**
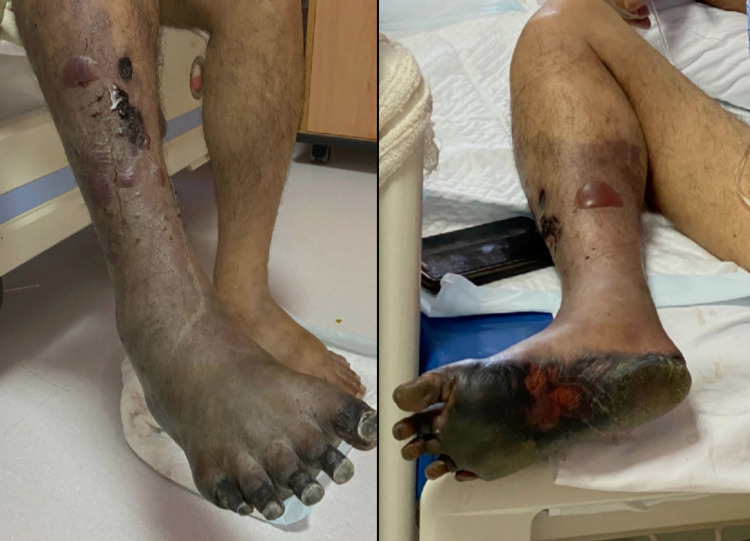
Right lower limb gangrene Right lower limb showing a black discoloration of the sole and dorsum of the foot with all toes (dry gangrene) associated with multiple swelling, bullae, and discharge in the leg with the demarcated line below the knee (wet gangrene)

On the second and third day, the vascular conditions of the right lower limb started to develop dry gangrene with increasing black discoloration day by day. Also, It was associated with severe pain. On hospital day five, the patient developed wet gangrene at the lower half of the right leg as well as dry gangrene of the right foot (Figure 3). The vascular surgery team was consulted again for the further management plan. Their impression was as the patient had a loss of sensation which is an absolute contraindication for any surgical intervention, embolectomy in this case will lead to reperfusion injury which has a high mortality rate. In addition, they recommend continuing on therapeutic dose anticoagulation and right above knee major amputation.

Day by day, the patient was improving concerning respiratory symptoms and oxygen requirement. On hospital day seven, his oxygen saturation was 97% on 3 L through a nasal cannula and 93% on room air. On post-admission day nine, a nasopharyngeal swab for COVID-19 was collected again that showed negative results after two days. As our hospital is a COVID-19 centre, the patient was transferred to King Khalid Hospital for right above knee major amputation.

## Discussion

The SARS-CoV-2 virus has a diverse systemic manifestation, yet its exact mechanism is still unclear. Healthcare providers carefully observe the wide variety of clinical pictures this virus may cause. Respiratory symptoms are more associated with the COVID-19 disease; however, the constitutional involvement of other systems such as cardiovascular, neurology, gastrointestinal tract, and hematology became more obvious [6,7]. In our patient, massive bilateral lower limbs ischemia has taken place, markedly apparent in the right limb resulting in wet gangrene of the lower leg and dry gangrene of the foot. We believe an acute thrombosis has occurred in a matter of days, which could be strongly linked to pro-thrombotic and inflammatory states associated with SARS-CoV-2 infection. The currently proposed mechanism behind systemic manifestation, mainly coagulation in our discussion, is the expression of angiotensin-converting enzyme 2 (ACE-2) receptors in various tissues including endothelial tissue [8]. The SAR-CoV-2 virus is evidenced to damage the endothelial cells, alter the receptors' normal mechanism, and activate coagulation cascades [4].

The hypercoagulable state is commonly seen in critically ill patients with COVID-19 [9,10]. In critical ICU patients, venous thromboembolism events (VTE) are more commonly noticed. Arterial thrombosis is considered a rare manifestation; however, arterial and venous thrombosis are considered poor prognostic factors especially in comorbid populations [5,9]. The incidence of acute limb ischemia (ALI) is around five times higher in COVID-19 positive cases when relatively compared to the usual number of 1.5 cases per 10,000 persons per year prior to the pandemic. In Brescia, Italy, the same study reported that ALI has increased from 1.8% to 16.3% during the COVID-19 pandemic in 2019 and 2020, respectively [11]. Bellosta et al. demonstrated a recent cohort study of 20 patients with a higher number of cases of ALI due to arterial thrombosis with confirmed COVID-19 cases [11]. Moreover, The derangement of hemostasis in patients with COVID-19 which leads to consumption of coagulation factors has a risk to develop disseminated intravascular coagulation (DIC), all of which have been linked to negative prognosis [4,12].

The etiology of arterial thrombosis is linked to the pro-inflammatory state associated with SARS-CoV-2 infection which is considered as the main contributor to hematologic dysfunction. The D-dimers level has been speculated as a prognostic parameter for thrombotic events associated with disease [13]. The same cohort study of 20 patients with ALI had a mean D-dimers of 2200 ng/L, which is much lower than in our case. Several markers have been described in the literature which are elevated with pro-inflammatory states such as CRP, ferritin, and interleukin(IL)-6. Other pro-thrombotic markers such as D-dimers, partial thromboplastin time (PTT), prothrombin time (PT), fibrinogen, fibrin degradation products (FDP), have also been defined [4]. However, clinical judgment to initiate prophylactic anticoagulation should be made irrespective of D-dimers level, as not all cases with arterial thrombosis have high D-dimers. Singh et al. reported a case series of three patients with COVID-19 who developed massive arterial thrombosis with inconsistent levels of D-dimers [14]. In addition, D-dimers and FDP were most notably extrapolative of disease progression; therefore, their routine surveillance would seem advisable in patients with COVID-19 [4,6].

Pre-existing peripheral arterial disease (PAD) should be screened and monitored closely in patients with comorbidities and has a diagnosis of SARS-CoV-2 infection as potential exacerbation to an acute thrombosis may arise. Moreover, anti-coagulation with low molecular weight heparin (LMWH) should be initiated in any severe COVID-19 admitted case with high D-dimers, as it appears to improve survival [15]. Interestingly, in our clinical scenario, despite the therapeutic dose of LMWH that has been commenced, the patient developed massive ischemia and his gangrene continue to deteriorate. Bellosta et al. concluded that the use of systemic heparin following revascularization improves treatment efficacy, limb salvage, and overall survival [11]. In our case, revascularization was not performed as irreversible ischemia has already ensued.

## Conclusions

COVID-19 is a newly emerging pandemic with a devastating number of cases worldwide. The virus predominantly affects the respiratory system; however, it evolved to affect multi-organs and causes systemic and coagulation dysfunctions mainly venous and arterial thrombosis. Several inflammatory markers that correlate with the degree of pro-thrombotic state have been speculated. In critically ill COVID-19 patients, arterial thrombosis should be monitored closely and anticoagulation therapy after revascularization should be commenced regardless of D-dimers level.
